# Spontaneous Exciton Collapse in a Strongly Flattened Ellipsoidal InSb Quantum Dot

**DOI:** 10.1186/s11671-022-03710-7

**Published:** 2022-08-23

**Authors:** K. G. Dvoyan, A. Karoui, B. Vlahovic

**Affiliations:** Department of Mathematics and Physics, North Carolina Central University, 1801 Fayetteville St., Durham, NC 27707 USA

**Keywords:** Klein–Gordon equation, Ellipsoidal quantum dot, Narrow band gap semiconductor, Adiabatic approximation, Kane’s dispersion law, Parabolic dispersion law, Exciton spontaneous collapse (decay), Coulomb accidental instability, Direct light absorption, Selection rules

## Abstract

Electronic and excitonic states in an *InSb* strongly flattened ellipsoidal quantum dot (QD) with complicated dispersion law are theoretically investigated within the framework of the geometric adiabatic approximation in the strong, intermediate, and weak quantum confinement regimes. For the lower levels of the spectrum, the square root dependence of energy on QD sizes is revealed in the case of Kane’s dispersion law. The obtained results are compared to the case of a parabolic (standard) dispersion law of charge carriers. The possibility of the accidental exciton instability is revealed for the intermediate quantum confinement regime. For the weak quantum confinement regime, the motion of the exciton's center-of-gravity is quantized, which leads to the appearance of additional Coulomb-like sub-levels. It is revealed that in the case of the Kane dispersion law, the Coulomb levels shift into the depth of the forbidden band gap, moving away from the quantum confined level, whereas in the case of the parabolic dispersion law, the opposite picture is observed. The corresponding selection rules of quantum transitions for the interband absorption of light are obtained. New selection rules of quantum transitions between levels conditioned by 2D exciton center of mass vertical motion quantization in a QD are revealed. The absorption threshold behavior characteristics depending on the QDs geometrical sizes are also revealed.

## Introduction

The achievements of modern semiconductor technologies provide ample opportunities for the design and production of the semiconductor QDs having nontrivial geometric shapes [1–8]. The energy spectrum of the QD is linear due to the complete quantization of charge carriers’ (CCs) motion, which makes it possible to call these structures artificial atoms. However, in contrast to real atoms, where the CC quantization is due solely to the Coulomb interaction, the discrete spectrum in the QD is formed due to quantum confinement, and in addition to charged particles (an electron, hole, impurity), uncharged particles (a phonon, exciton, biexciton) are affected by the quantum confinement as well. Another advantage of the quantum confinement is that by changing the size, external shape, or material of the QD, one can successfully control the energy spectrum of CCs in them. Obviously, an increase in the number of the QD geometric parameters leads to the possibility of more flexible and effective control of the energy spectrum and other physical characteristics of CCs in them. For example, Hund's rule is fulfilled for spherical QDs, whereas for ellipsoidal QDs this rule becomes more complicated, namely it leads to the appearance of new rules for filling the electronic shells and to the partial cancellation of old rules [4].

In low-dimensional structures, quantum confinement successfully competes with Coulomb quantization and even prevails over it in certain cases. The significant difference in the effective masses of an impurity (hole) and electron allows application of the Born–Oppenheimer approximation [9, 10]. When the quantum confined energy is much more than the Coulomb energy, the perturbation theory is applicable, where the role of a small correction plays the term of the Coulomb interaction in the problem Hamiltonian [10]. The situation is radically changed when the effective mass of the impurity center (hole) is comparable to the mass of the electron. For example, in the narrow-gap semiconductors for which the CC standard (parabolic) dispersion law is violated, the effective masses of the electron and light hole are equal [11–13], and obviously, Born–Oppenheimer approximation is not further applicable.

In recent years, the most unexpected areas of application of QDs’ properties have appeared in the design of new high-tech devices used in science, technology, medical, and domestic appliances fields. On the basis of QDs, quantum lasers, LEDs of various spectra [14–16], biochemical sensors [17, 18], single-electron diodes and transistors [19, 20], and saturated TV screens with a wide range of different colors have been designed and successfully implemented. Recent studies also show that QDs are key objects for the successful implementation of qubits [21–23]. One of the priority tasks of modern semiconductor physics is the development of a new generation of label-free biochemical sensors based on quantum confinement and tunneling between nanostructures as well as sensors for detection of structural changes in the material. Currently existing biochemical sensors are based, for example, on the chemical capture of surrounding molecules using ligands. However, this method allows only one-time use of the sensor, since after the formation of a chemical bond, the sensor becomes inoperative (unusable) or needs to be cleaned. For sensors based on the quantum tunneling effect, such issues are eliminated, since chemical bonds do not arise, and the detection of molecules occurs only on the basis of electron tunneling. It should be noted that the tunneling of electrons from analyte to the QD is possible only if the electronic levels of the QD and analyte coincide. In addition to energy orbitals, analyte molecules also have families of vibration and rotation levels, the interlevel distances of which are a kind of unique markers (fingerprints). For successful imitation of these families of levels, QDs with a specific external shape—strongly flattened QDs—are suitable, which ensures the appearance of similar families of levels within the QD. For a more successful mimicry of inter-level distances, the correct choice of QD material is also important. For this very purpose, in this work, we consider the electronic properties, exciton states, and direct interband absorption of light in a strongly flattened ellipsoidal QD with the Kane dispersion law of CCs. Theoretical calculations on the spectra of these systems will provide wide possibilities for modeling of highly selective quantum sensors and highly sensitive structural sensors of the new generation, with a wide range of controllable properties.

## Theory

**Fig. 1:**
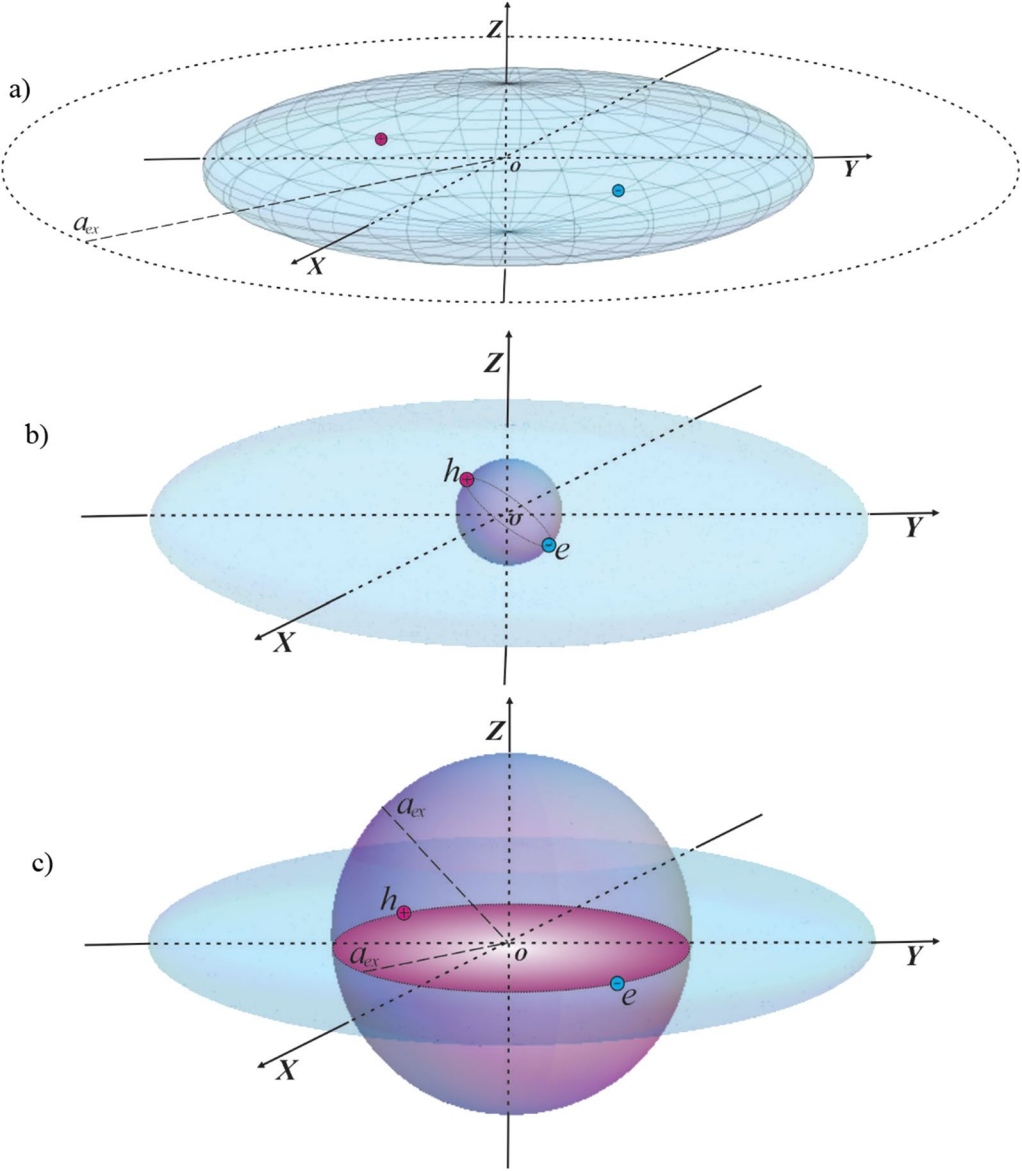
Strongly flattened ellipsoidal QD: (**a**) realization of the strong quantum confinement regime, when the condition $a_{ex} > > a > > c$ holds, and the motions of an electron and a hole are quantized separately, (**b**) realization of the weak quantum confinement regime, when the condition $c > > a_{ex}$ holds, and the motion of a 3D exciton is quantized, (**c**) realization of the intermediate quantum confinement regime, when the condition $a > > a_{ex} > > c$ holds, and the motion of a 2D exciton is quantized

Let us consider an impermeable strongly flattened ellipsoidal QD (see Fig. 1.). Then, the potential energy of the CC in cylindrical coordinates can be written in the following form:

1 $$U\left( {\rho ,\varphi ,Z} \right) = \left\{ \begin{gathered} 0,\,\,\frac{{\rho^{2} }}{{a_{1}^{2} }} + \frac{{Z^{2} }}{{c_{1}^{2} }} \le 1 \hfill \\ \infty ,\,\,\frac{{\rho^{2} }}{{a_{1}^{2} }} + \frac{{Z^{2} }}{{c_{1}^{2} }} > 1 \hfill \\ \end{gathered} \right.,\,\,\,a_{1} > > c_{1}$$

where $a_{1}$ and $c_{1}$ are minor and major semiaxes of the ellipsoid, respectively. One needs to compare the geometric sizes of the QD with effective exciton radius of the CC in order to determine the quantum confinement regimes.

### Strong Quantum Confinement Regime

First, we solve the problem in the strong quantum confinement regime, when the condition $a_{ex} > > a_{1} > > c_{1}$ takes place (see Fig. 1a), and $a_{ex}$ is the effective Bohr radius of an exciton. In this approximation, the Coulomb interaction between an electron and a hole is much less than the quantum confinement energy, therefore the former can be neglected. Then, the problem reduces to the determination of CCs’ energy states independently. As noted above, the dispersion law for narrow-gap semiconductors is non-parabolic and is given in the following form [11, 24]:

2 $$E^{2} = P^{2} S^{2} + m_{e(h)}^{*\,2} S^{4}$$

where $S \sim 10^{8} sm/\sec$ is the parameter related with the semiconductor band gap $E_{g} = 2m_{e}^{*} S^{2}$. Let us write the Klein–Gordon equation [25] for an ellipsoidal QD consisting of ${\text{InSb}}$, with an electron and hole when their Coulomb interaction is neglected:

3 $$\sqrt {\left( {P_{e}^{2} + P_{h}^{2} } \right)S^{2} + \left( {m_{e}^{*\,2} + m_{h}^{*\,2} } \right)S^{4} } \,\Psi \left( {\vec{r}_{e} ,\vec{r}_{h} } \right) = E\,\Psi \left( {\vec{r}_{e} ,\vec{r}_{h} } \right)$$

Here $P_{e(h)}$ is the momentum operator of the CC (electron, hole), $m_{e(h)}^{*}$ is the effective mass of the CC, and $E$ is the total energy of the system. After simple transformations, equation (3) can be written as the reduced Schrödinger equation in dimensionless units:

4 $$\left( { - \frac{1}{2}\nabla_{e}^{2} - \frac{1}{2}\nabla_{h}^{2} } \right)\Psi \left( {\vec{r}_{e} ,\vec{r}_{h} } \right) = \varepsilon_{0} \Psi \left( {\vec{r}_{e} ,\vec{r}_{h} } \right)$$

where $\varepsilon_{0} = \frac{{2\varepsilon^{2} - \varepsilon_{g}^{2} }}{{2\varepsilon_{g} }},\,\,\varepsilon = \frac{E}{{E_{ex} }},\,\,\varepsilon_{g} = \frac{{E_{g} }}{{E_{ex} }}$, $E_{ex} = \frac{{\hbar^{2} }}{{2\mu a_{ex}^{2} }} = \frac{{e^{2} }}{{\kappa a_{ex} }}$ is the effective Rydberg energy of an exciton, $\kappa$ is the dielectric constant of the semiconductor, $a_{ex} = \frac{{\kappa \hbar^{2} }}{{\mu e^{2} }}$ is an exciton effective Bohr radius, $\mu = \frac{{m_{e}^{*} m_{h}^{*} }}{{m_{e}^{*} + m_{h}^{*} }}$ is the reduced mass of an exciton, and $e$ is the elementary charge. The wave functions (WFs) of the problem are sought in the form $\Psi \left( {\vec{r}_{e} ,\vec{r}_{h} } \right) = \Psi_{e} \left( {\vec{r}_{e} } \right)\Psi_{h} \left( {\vec{r}_{h} } \right)$. After separation of variables, one can obtain the following equation for the electron:

5 $$\left( {\nabla_{e}^{2} + 2\varepsilon_{e} } \right)\Psi_{e} \left( {\vec{r}_{e} } \right) = 0$$

The CC motion in the radial direction is much slower than in the direction $OZ$ due to the geometric shape of the QD ($a_{1} > > c_{1}$). Based on this, the Hamiltonian in the dimensionless variables can be represented as the sum of the Hamiltonians of the “fast” $\hat{H}_{1}$ and “slow” $\hat{H}_{2}$ subsystems [26, 27]:

6 $$\hat{H} = \hat{H}_{1} + \hat{H}_{2} + U\left( {r,\varphi ,z} \right)$$

where

7 $$\hat{H}_{1} = - \frac{{\partial^{2} }}{{\partial z^{2} }},\,\,\,\,\hat{H}_{2} = - \left( {\frac{{\partial^{2} }}{{\partial r^{2} }} + \frac{1}{r}\frac{\partial }{\partial r} + \frac{1}{{r^{2} }}\frac{{\partial^{2} }}{{\partial \varphi^{2} }}} \right)$$

Here, $\hat{H} = \frac{{\hat{\rm H}}}{{E_{ex} }}$, $r = \frac{\rho }{{a_{ex} }}$, $z = \frac{Z}{{a_{ex} }}$. The WFs are sought in the form:

8 $$\Psi_{e} \left( {r,\varphi ,z} \right) = Ce^{im\varphi } \chi \left( {z;r} \right)R\left( r \right)$$

where $C$ is the normalization constant. For a fixed value of the coordinate $r$ of the slow subsystem, the electron motion is localized in the one-dimensional potential well with an effective variable width:

9 $$L\left( r \right) = 2c\sqrt {1 - \frac{{r^{2} }}{{a^{2} }}}$$

where $a = \frac{{a_{1} }}{{a_{ex} }}$ and $c = \frac{{c_{1} }}{{a_{ex} }}$ notations are introduced. First, let us solve the Schrödinger equation for the “fast” subsystem, which can be written in the form of the harmonic equation:

10 $$\chi^{\prime\prime}\left( {z;r} \right) + \varepsilon \left( r \right)\chi \left( {z;r} \right) = 0$$

where $\varepsilon \left( r \right)$ is the energy of the “fast” subsystem. The solutions of Eq. (10) are given in the form:

11 $$\chi \left( {z;r} \right) = \sqrt {\frac{2}{L\left( r \right)}} \sin \left( {\frac{\pi n}{{L\left( r \right)}}z + \frac{\pi n}{2}} \right)$$

where $n$ is the quantum number (QN) of the “fast” subsystem. One can obtain the “fast” subsystem energy from the boundary conditions $\left. {\chi \left( {z;r} \right)} \right|_{{z = \pm \frac{L\left( r \right)}{2}}} = 0$, taking into account the expression (9):

12 $$\varepsilon \left( r \right) = \frac{{\pi^{2} n^{2} }}{{L^{2} \left( r \right)}},\,\,\,\,n = 1,2,...$$

For the lower levels of the energy spectrum, the electron motion is mainly localized in the region of the geometric center-of-gravity of the QD ($r < < a$). Based on this, one can expand in series $\varepsilon\left( r \right)$:

13 $$\varepsilon \left( r \right) \approx \varepsilon_{n}^{0} + \omega_{n}^{2} r^{2}$$

where $\varepsilon_{n}^{0} = \frac{{\pi^{2} n^{2} }}{{4c^{2} }}$ and $\omega_{n} = \frac{\pi n}{{2ac}}$ notations are introduced. Now let us consider the CC motion in the “slow” subsystem, for which the expression (13) serves as an effective potential energy. The Schrödinger equation of the “slow” subsystem takes the form:

14 $$\left( { - \left( {\frac{{\partial^{2} }}{{\partial r^{2} }} + \frac{1}{r}\frac{\partial }{\partial r} + \frac{1}{{r^{2} }}\frac{{\partial^{2} }}{{\partial \varphi^{2} }}} \right) + \varepsilon_{n}^{0} + \omega_{n}^{2} r^{2} } \right)R\left( r \right)e^{im\varphi } = 2\varepsilon_{e} \,R\left( r \right)e^{im\varphi }$$

After the change of a variable $\xi = \omega_{n} {\kern 1pt} r^{2}$ and $\gamma = \frac{{2\varepsilon_{e} - \varepsilon_{n}^{0} }}{{4\omega_{n} }}$ notation, Eq. (14) is written as

15 $$\xi R^{\prime\prime}\left( \xi \right) + R^{\prime}\left( \xi \right) + \left( { - \frac{{m^{2} }}{4\xi } + \gamma - \frac{\xi }{4}} \right)R\left( \xi \right) = 0$$

The solution of Eq. (15) is sought in the form of $R\left( \xi \right) \sim e^{{ - \frac{\xi }{2}}} \xi^{{\frac{\left| m \right|}{2}}} \Omega \left( \xi \right)$, after which the Kummer equation is obtained:

16 $$\xi \,\Omega^{\prime\prime}\left( \xi \right) + \left( {\left| m \right| + 1 - \xi } \right)\Omega^{\prime}\left( \xi \right) + \left( {\gamma - \frac{\left| m \right| + 1}{2}} \right)\Omega \left( \xi \right) = 0$$

the solutions of which are given by degenerate hypergeometric functions of the first kind:

17 $$\Omega \left( \xi \right) = {}_{1}F_{1} \left( { - \left( {\gamma - \frac{\left| m \right| + 1}{2}} \right),\left| m \right| + 1,\xi } \right)$$

For the total energy of an electron, from the boundary conditions, one obtains

18 $$\varepsilon_{e} = \frac{{\pi^{2} n^{2} }}{{8c^{2} }} + \frac{\pi n}{{2ac}}\left( {2n_{r} + \left| m \right| + 1} \right) = \frac{{\pi^{2} n^{2} }}{{8c^{2} }} + \frac{\pi n}{{2ac}}\left( {N + 1} \right)$$

where $n_{r} ,\,m$ and $N = 2n_{r} + \left| m \right|$ are the radial, magnetic, and oscillatory QNs of an electron, respectively. The electron energy (18) is a constant of separation of variables in the hole reduced Schrödinger equation:

19 $$\left( {\nabla_{h}^{2} + 2\left( {\varepsilon_{0} - \varepsilon_{e} } \right)} \right)\Psi_{h} \left( {\vec{r}_{h} } \right) = 0$$

Solving equation (19) in a similar way, in the strong quantum confinement regime, one can derive the following expression for the total energy of the particles’ system:

20 $$\varepsilon_{str}^{Kane} = \sqrt {\varepsilon_{g} } \sqrt {\frac{{\pi^{2} \left( {n^{2} + n^{{\prime}{2}} } \right)}}{{8c^{2} }} + \frac{\pi }{2ac}\left( {n\left( {2n_{r} + \left| m \right| + 1} \right) + n^{\prime}\left( {2n^{\prime}_{r} + \left| {m^{\prime}} \right| + 1} \right)} \right) + \frac{{\varepsilon_{g} }}{2}} \,$$

Here, $n,n_{r} ,\,m$ and $n^{\prime},n^{\prime}_{r} ,\,m^{\prime}$ are the QNs of electron and hole, respectively. For comparison (see (20)), in the case of a parabolic dispersion law (e.g., for QD consisting of $GaAs$) the total energy in the strong quantum confinement regime is given as [5]:

21 $$\varepsilon_{str}^{par} = \frac{{\pi^{2} n^{2} }}{{8c^{2} }} + \frac{\pi n}{{2ac}}\left( {N + 1} \right) + \frac{{\pi^{2} n^{{\prime}{2}} }}{{8c^{2} }} + \frac{{\pi n^{\prime}}}{2ac}\left( {N^{\prime} + 1} \right),\,\,\,\,N,N^{\prime} = 0,1,2,...$$

Here, $n,N$ and $n^{\prime},N^{\prime}$ are the QNs of “fast” and “slow” subsystems of electron and hole, respectively. Normalized WFs are given in the form:

22 $$\begin{gathered} \,\,\,\Psi_{e\left( h \right)} \left( {r,\varphi ,z} \right) = \frac{{e^{im\varphi } }}{{\sqrt {2\pi } }}\sqrt {\frac{2}{L\left( r \right)}} \sin \left( {\frac{\pi n}{{L\left( r \right)}}z + \frac{\pi n}{2}} \right) \times \hfill \\ \times \sqrt {\frac{\pi n}{{ac}}} \frac{{\sqrt {n_{r} !} \Gamma \left( {\left| m \right| + 1} \right)}}{{\Gamma^{{{3 \mathord{\left/ {\vphantom {3 2}} \right. \kern-\nulldelimiterspace} 2}}} \left( {\left| m \right| + 1 + n_{r} } \right)}}e^{{ - \frac{\pi n}{{2ac}}r^{2} }} \left( {\frac{\pi n}{{2ac}}r^{2} } \right)^{{\frac{\left| m \right|}{2}}} {}_{1}F_{1} \left\{ { - n_{r} ,\left| m \right| + 1;\frac{\pi n}{{2ac}}r^{2} } \right\}. \hfill \\ \end{gathered}$$

### Weak Quantum Confinement Regime

Let us discuss the weak quantum confinement regime, when the condition $a_{ex} < < c_{1}$ is satisfied (see Fig. 1b). Then, the binding energy of the exciton prevails over the quantum confinement energy, and the weak influence of the QD walls appears as a small correction. In other words, the quantized motion of an exciton as a whole is considered in a strongly flattened ellipsoidal QD. In the case of the presence of Coulomb interaction between an electron and hole, the Klein–Gordon equation can be written as [25, 28–30]

23 $$\sqrt {\left( {\left( {P_{e}^{2} + P_{h}^{2} } \right)S^{2} + \left( {m_{e}^{*\,2} + m_{h}^{*\,2} } \right)S^{4} } \right)} \Psi \left( {\vec{r}_{e} ,\vec{r}_{h} } \right) = \left( {E + \frac{{e^{2} }}{{\kappa \left| {\vec{r}_{e} - \vec{r}_{h} } \right|}}} \right)\Psi \left( {\vec{r}_{e} ,\vec{r}_{h} } \right)$$

After some transformations, as in the case of a strong quantum confinement regime, the Klein–Gordon equation reduces to the Schrödinger equation with a certain effective energy. Using the coordinates of the exciton’s center-of-gravity $\vec{r} = \vec{r}_{e} - \vec{r}_{h}$, $\vec{R} = \frac{{m_{e}^{ * } \vec{r}_{e} + m_{h}^{ * } \vec{r}_{h} }}{{m_{e}^{ * } + m_{h}^{ * } }}$, where $\vec{r}_{e}$ and $\vec{r}_{h}$ are the 3D radius-vectors of an electron and a hole, respectively, $m_{h}^{ * }$ is the effective mass of a hole, and considering the case of a light hole $m_{e}^{ * } = m_{h}^{ * }$, one can represent the system WFs in the following form:

24 $$\Psi \left( {\vec{r}_{e} ,\vec{r}_{h} } \right) = \psi_{{n_{r} ,l,q}} \left( {\vec{r}} \right)\Phi_{{n_{Gr} ,n_{R} ,M}} \left( {\vec{R}} \right)$$

Here, the WF $\psi_{{n_{r} ,l,q}} \left( {\vec{r}} \right)$ describes the relative motion of an electron and a hole, and WF $\Phi_{n,m,k} \left( {\vec{R}} \right)$ describes the motion of the exciton’s center-of-gravity, where $n_{r} ,\,l,\,q$ are the radial, orbital, and magnetic QNs of the exciton, correspondingly. After switching to the new coordinates, the reduced Schrödinger equation takes the following form:

25 $$\left( { - \frac{{\hbar^{2} }}{{2M_{0} }}\nabla_{{\vec{R}}}^{2} - \frac{{\hbar^{2} }}{2\mu }\nabla_{{\vec{r}}}^{2} } \right)\psi_{{n_{r} ,l,q}} \left( {\vec{r}} \right)\Phi_{{n_{Gr} ,n_{R} ,M}} \left( {\vec{R}} \right) = \left( {\frac{{\left( {E + \frac{{e^{2} }}{{\kappa \left| {\vec{r}} \right|}}} \right)^{2} - \left( {m_{e}^{*\,2} + m_{h}^{*\,2} } \right)S^{4} }}{{2m_{e}^{ * } S^{2} }}} \right)\psi_{{n_{r} ,l,q}} \left( {\vec{r}} \right)\Phi_{{n_{Gr} ,n_{R} ,M}} \left( {\vec{R}} \right)$$

where $M_{0} = m_{e}^{ * } + m_{h}^{ * }$ is the mass of an exciton. In the $E_{ex}$ and $a_{ex}$ units, Eq. (25)  is written in the form:

26 $$\left( { - \frac{1}{4}\nabla_{{\vec{R}}}^{2} - \nabla_{{\vec{r}}}^{2} } \right)\psi_{{n_{r} ,l,q}} \left( {\vec{r}} \right)\Phi_{{n_{Gr} ,n_{R} ,M}} \left( {\vec{R}} \right) = \left( {\varepsilon_{0} + \frac{\alpha }{r} + \frac{\beta }{{r^{2} }}} \right)\psi_{{n_{r} ,l,q}} \left( {\vec{r}} \right)\Phi_{{n_{Gr} ,n_{R} ,M}} \left( {\vec{R}} \right)$$

where $\varepsilon_{0} = \frac{{2\varepsilon^{2} - \varepsilon_{g}^{2} }}{{2\varepsilon_{g} }},\,\,\alpha = \frac{4\varepsilon }{{\varepsilon_{g} }},\,\,\beta = \frac{4}{{\varepsilon_{g} }}$ notations are introduced. One can derive the equation for the exciton’s center-of-gravity, after separation of variables:

27 $$- \frac{1}{4}\nabla_{{\vec{R}}}^{2} \Phi_{{n_{Gr} ,n_{R} ,M\left( {N_{R} } \right)}} \left( {\vec{R}} \right) = \varepsilon_{Gr} \Phi_{{n_{Gr} ,n_{R} ,M\left( {N_{R} } \right)}} \left( {\vec{R}} \right)$$

The energy $\varepsilon_{Gr}$ of the exciton’s center-of-gravity can be obtained by repeating the procedure of calculations of the strong quantum confinement regime for the adiabatic approximation, considering the exciton mass $M_{0}$ instead of the $m_{e}^{ * }$:

28 $$\varepsilon_{Gr} = \frac{{\pi^{2} n_{Gr}^{2} }}{{16c^{2} }} + \frac{{\pi n_{Gr} }}{4ac}\left( {2n_{R} + \left| M \right| + 1} \right) = \frac{{\pi^{2} n_{Gr}^{2} }}{{16c^{2} }} + \frac{{\pi n_{Gr} }}{4ac}\left( {N_{R} + 1} \right)$$

where $n_{Gr}$ is the QN of the “fast” subsystem of exciton’s center-of-gravity motion, $n_{R} ,\,M$, and $N_{R} = 2n_{R} + \left| M \right|$ are the radial, magnetic, and oscillatory QNs of the “slow” subsystem of the same motion, respectively.

Further, let us consider the relative motion of the electron–hole pair. The WFs of the problem are sought in the form $\psi_{{n_{r} ,l,q}} \left( {\vec{r}} \right) = \frac{1}{\sqrt r }{\rm X}_{{n_{r} ,l}} \left( r \right)Y_{lq} \left( {\theta ,\varphi } \right)$, where $Y_{lq} \left( {\theta ,\varphi } \right)$ are spherical functions, $n_{r} ,\,\,l,\,\,q$ are radial, orbital, and magnetic QNs of relative motion. After simple transformations, the radial part of the reduced Schrödinger equation can be written as:

29 $${\rm X}^{\prime\prime}\left( r \right) + \frac{1}{r}{\rm X}^{\prime}\left( r \right) + \left( {\varepsilon_{1} - \frac{{\left( {l + \frac{1}{2}} \right)^{2} - \beta }}{{r^{2} }} + \frac{\alpha }{r}} \right){\rm X}\left( r \right) = 0$$

where $\varepsilon_{1} = \varepsilon_{0} - \varepsilon_{Gr}$. The change of variable $\eta = 2\sqrt { - \varepsilon_{1} } r$ transforms Eq. (29) to

30 $${\rm X}^{\prime\prime}\left( \eta \right) + \frac{1}{\eta }{\rm X}^{\prime}\left( \eta \right) + \left( { - \frac{1}{4} - \frac{{\left( {l + \frac{1}{2}} \right)^{2} - \beta }}{{\eta^{2} }} + \frac{\delta }{\eta }} \right){\rm X}\left( \eta \right) = 0$$

where the parameter $\delta = \frac{\alpha }{{2\sqrt { - \varepsilon_{1} } }}$ is introduced. When $\eta \to 0$, the desired solution of (30) is sought in the form ${\rm X}\left( {\eta \to 0} \right) = {\rm X}_{0} \sim \eta^{\lambda }$[29]. Substituting this in Eq. (30), one gets a quadratic equation with two solutions:

31 $$\lambda_{1,2} = \mp \sqrt {\left( {l + \frac{1}{2}} \right)^{2} - \beta }$$

The solution satisfying the finiteness condition of the WF is given as $${\rm X}_{0} \sim \eta^{{\sqrt {\left( {l + \frac{1}{2}} \right)^{2} - \beta } }}$$. When $$\eta \to \infty$$, equation (30) takes the form: $${\rm X}^{\prime\prime}\left( \eta \right) - \frac{1}{4}{\rm X}\left( \eta \right) = 0$$. The solution satisfying the standard conditions can be written as $${\rm X}\left( {\eta \to \infty } \right) = {\rm X}_{\infty } \sim e^{{ - {\raise0.7ex\hbox{$\eta $} \!\mathord{\left/ {\vphantom {\eta 2}}\right.\kern-\nulldelimiterspace} \!\lower0.7ex\hbox{$2$}}}}$$ [28, 29]. Thus, the solution is sought in the form:

32 $${\rm X}\left( \eta \right) = \eta^{\lambda } e^{{ - {\raise0.7ex\hbox{$\eta $} \!\mathord{\left/ {\vphantom {\eta 2}}\right.\kern-\nulldelimiterspace} \!\lower0.7ex\hbox{$2$}}}} f\left( \eta \right)$$

Substituting the function (32) into Eq. (30) one gets the Kummer equation [30]:

33 $$\eta f^{\prime\prime}\left( \eta \right) + \left( {2\lambda + 1 - \eta } \right)f^{\prime}\left( \eta \right) + \left( {\delta - \lambda - \frac{1}{2}} \right)f\left( \eta \right) = 0$$

the solutions of which are given by the first kind degenerate hypergeometric functions:

34 $$f\left( \eta \right) =_{1} F_{1} \left( { - \left( {\delta - \lambda - \frac{1}{2}} \right),2\lambda + 1,\eta } \right)$$

The expression $\delta - \lambda - \frac{1}{2}$ needs to be a nonnegative integer $n_{r}$ (radial QN) providing the finiteness of the WFs:

35 $$n_{r} = \delta - \lambda - \frac{1}{2},\,\,\,\,\,\,\,n_{r} = 0,1,2,....$$

From the condition (35) for the energy, one can derive the following expression in dimensionless units:

36 $$\varepsilon_{weak}^{Kane} = - \frac{{\sqrt {\frac{{\varepsilon_{g} }}{2} + \varepsilon_{Gr} } }}{{\sqrt {\frac{1}{{\varepsilon_{g} }} + \frac{4}{{\varepsilon_{g}^{2} \left( {n_{r} + \sqrt {\left( {l + \frac{1}{2}} \right)^{2} - \frac{4}{{\varepsilon_{g} }}} + \frac{1}{2}} \right)^{2} }}} }}$$

or

37 $$\varepsilon_{weak}^{Kane} = - \frac{{\sqrt {\frac{{\varepsilon_{g} }}{2} + \frac{{\pi^{2} n_{Gr}^{2} }}{{16c^{2} }} + \frac{{\pi n_{Gr} }}{4ac}\left( {2n_{R} + \left| M \right| + 1} \right)} }}{{\sqrt {\frac{1}{{\varepsilon_{g} }} + \frac{4}{{\varepsilon_{g}^{2} \left( {n_{r} + \sqrt {\left( {l + \frac{1}{2}} \right)^{2} - \frac{4}{{\varepsilon_{g} }}} + \frac{1}{2}} \right)^{2} }}} }}$$

For comparison, in the case of parabolic dispersion law, system energy of the weak confinement regime is given by the formula

38 $$\varepsilon_{weak}^{par} = \frac{{\pi^{2} n_{Gr}^{2} }}{{16c^{2} }} + \frac{{\pi n_{Gr} }}{4ac}\left( {2n_{R} + \left| M \right| + 1} \right) - \frac{1}{{N_{C}^{2} }} = \frac{{\pi^{2} n_{Gr}^{2} }}{{16c^{2} }} + \frac{{\pi n_{Gr} }}{4ac}\left( {N_{R} + 1} \right) - \frac{1}{{N_{C}^{2} }}$$

where $N_{C} = 1,2,...$ is the Coulomb main QN of the exciton.

It is necessary to note some important results:

- In contrast to the case of the problem of hydrogen impurities in a semiconductor with Kane’s dispersion law, considered in works [31, 32], in the case of the exciton, the instability of the ground state energy is absent. Thus, in the case of hydrogen-like impurity, the electron energy becomes unstable when $Z\alpha_{0} > \frac{1}{2}$ ($Z$ is an ordinal number, $\alpha_{0}$ is the fine structure constant), and the phenomenon of the particle falling into the center takes place. However, in the case of an exciton with Kane’s dispersion law, the expression $\left( {l + \frac{1}{2}} \right)^{2} - \frac{4}{{\varepsilon_{g} }}$ under the square root does not become negative even for the ground state with $l = 0$, hence fulfillment of the condition $\alpha_{0} > \frac{1}{\sqrt 2 }$ would be necessary to obtain instability in the ground state.
- For narrow band gap semiconductor QDs, the quantum confined motion introduces an energy term under the square root in the energy of an exciton (expressed in the center-of-gravity referential), whereas in the case of parabolic dispersion law, this energy appears as a linear expression (a simple sum).
- The exciton energy depends only on the main QN of the Coulomb motion in the case of realization of the parabolic dispersion, whereas in the case of Kane’s dispersion law it reveals a rather complicated dependence on the radial and orbital QNs. Thus, the non-parabolicity of the charge carrier’s dispersion law leads to the removal of “random” Coulomb degeneracy in the orbital QN.
- In the case of the implementation of the parabolic dispersion law, a family of Coulomb levels is located under each quantum confined level; with an increase in the Coulomb QN, these levels shift closer to the quantum confined level (see 38). A similar situation is observed in the case of a diamagnetic exciton[33], where the magnetic quantization energy level, under which the Coulomb levels are located, takes the quantum confined level role. The picture is the opposite in the case of the implementation of the Kane’s dispersion law. In a narrow-gap semiconductor, due to interband interaction, the quantum confined and Coulomb energies do not appear as additive terms in the total energy, but as a sum under the square root (see 37). In this case, with an increase in the Coulomb QNs, the levels shift deeper into the forbidden band, moving away from the quantum confined level. This consolidation of Coulomb levels resembles the behavior of acceptor levels, while in the case of a parabolic dispersion law, exciton levels behave similarly to donor levels.

### Intermediate Quantum Confinement Regime ($a > > a_{ex} > > c$)

Let us discuss the intermediate-weak quantum confinement regime, when the condition $a > > a_{ex} > > c$ is satisfied (see Fig. 1c). In this regime, in the $OZ$-direction, the quantum confinement significantly exceeds the Coulomb interaction of an electron and a hole, however, in the radial direction the picture is the opposite. In this case, the system’s energy for ***the radial motion*** is caused mainly by the electron–hole Coulomb interaction, and the formation of quasi-2D exciton is possible. Note that such a quantization regime was not considered in the case of a parabolic dispersion law either. Repeating the calculation procedure as in the case of a weak quantum confinement regime, we switch to the coordinates of the center-of-gravity and relative motion. Since the motion of the center-of-gravity is the motion of an electrically neutral particle, there is no competition for this motion between quantum confinement and Coulomb quantization. Again, one solves the Klein–Gordon equation, and repeat the calculations from (23) to (28). Further, let us consider in more detail the relative motion of an electron and a hole. In the case of wide-gap semiconductors, the probability of the formation of excitons with a heavy hole more often prevails over the probability of the formation of an exciton with a light hole due to the specificity of the band structure. Therefore, in the case of an intermediate quantum confinement regime, the motion of a heavier particle, a hole, is considered in the averaged field created by a faster and lighter particle, an electron. In other words, the Born–Oppenheimer approximation is used to describe the motion of a heavy hole in a potential field created by an electron. To solve this problem, the electron potential is expanded in the Taylor series at the coordinate of the hole motion. In  this case, when a light hole is considered, such an approach would be completely unjustified. Considering the condition $a_{ex} > > c_{1}$, we can assert that the motion of a quasi-2D exciton is ensured under the condition $z < < r$. Therefore, instead of the averaged potential, we expand the Hamiltonian of the relative motion in a series, omitting the terms proportional to $\frac{{z^{2} }}{{r^{2} }} \to 0$. In other words, let us consider the relative motion of a quasi-2D exciton, which is confined in the perpendicular direction by the walls of the QD. Then, in cylindrical coordinates, the reduced Schrödinger equation for relative motion will be written in the form:

39 $$\left( {\frac{{\partial^{2} }}{{\partial z^{2} }} + \frac{{\partial^{2} }}{{\partial r^{2} }} + \frac{1}{r}\frac{\partial }{\partial r} + \frac{1}{{r^{2} }}\frac{{\partial^{2} }}{{\partial \varphi^{2} }} + \left( {\varepsilon_{0} - \varepsilon_{Gr} } \right) + \frac{\alpha }{r} + \frac{\beta }{{r^{2} }}} \right)\psi_{{n_{r} ,m,n_{z} }} \left( {r,\varphi ,z} \right) = 0$$

WFs are sought in the form of

40 $$\psi_{{n_{r} ,m,n_{z} }} \left( {r,\varphi ,z} \right) = Ce^{im\varphi } {\rm X}_{{n_{r} ,m}} \left( r \right)D_{{n_{z} }} \left( z \right)$$

where $C$ is the normalization constant. After separating the variables, for the WFs and the energy of motion in the $OZ$-direction, respectively, one gets:

41 $$D_{{n_{z} }} \left( z \right) = \sqrt {\frac{a}{{c\sqrt {a^{2} - 1} }}} \sin \left( {\frac{{\pi \,a\,n_{z} }}{{2c\sqrt {a^{2} - 1} }}z + \frac{{\pi n_{z} }}{2}} \right),\,\,\,n_{z} = 1,2,...$$

42 $$\varepsilon_{z} = \frac{{\pi^{2} n_{z}^{2} a^{2} }}{{4c^{2} \left( {a^{2} - 1} \right)}}$$

Further, for the radial part, one obtains equation

43 $${\rm X}^{\prime\prime}\left( r \right) + \frac{1}{r}{\rm X}^{\prime}\left( r \right) + \left( {\varepsilon_{2} - \frac{{m^{2} - \beta }}{{r^{2} }} + \frac{\alpha }{r}} \right){\rm X}\left( r \right) = 0$$

where the notation $\varepsilon_{2} = \varepsilon_{0} - \varepsilon_{Gr} - \varepsilon_{z}$ is introduced. The change of variable $\xi = 2\sqrt { - \varepsilon_{2} } r$ transforms Eq. (29) to

44 $${\rm X}^{\prime\prime}\left( \xi \right) + \frac{1}{\xi }{\rm X}^{\prime}\left( \xi \right) + \left( { - \frac{1}{4} - \frac{{m^{2} - \beta }}{{\xi^{2} }} + \frac{\delta }{\xi }} \right){\rm X}\left( \xi \right) = 0$$

where the similar parameter $\delta = \frac{\alpha }{{2\sqrt { - \varepsilon_{2} } }}$ is introduced.

At $\xi \to 0$ the solution of (44) is sought in the form $\chi \left( {\xi \to 0} \right) = \chi_{0} \sim \xi^{\lambda }$. Here, in contrast to Eq. (30) the quadratic equation is obtained with the following solutions:

45 $$\lambda_{1,2} = \mp \sqrt {m^{2} - \beta }$$

In 2D case, the solution satisfying the condition of finiteness of the WF is given as $\chi_{0} \sim \xi^{{\sqrt {m^{2} - \beta } }}$. At $\xi \to \infty$, proceeding analogously to the solution of Eq. (30), one should again arrive at the equation of Kummer (33), but with different parameter $\lambda$. Finally, for the energy of the 2D exciton with the Kane dispersion law one gets:

46 $$\varepsilon_{int}^{Kane} = - \frac{{\sqrt {\frac{{\varepsilon_{g} }}{2} + \frac{{\pi^{2} n_{Gr}^{2} }}{{16c^{2} }} + \frac{{\pi n_{Gr} }}{4ac}\left( {2n_{R} + \left| M \right| + 1} \right) + \frac{{\pi^{2} n_{z}^{2} a^{2} }}{{4c^{2} \left( {a^{2} - 1} \right)}}} }}{{\sqrt {\frac{1}{{\varepsilon_{g} }} + \frac{4}{{\varepsilon_{g}^{2} \left( {n_{r} + \sqrt {m^{2} - \frac{4}{{\varepsilon_{g} }}} + \frac{1}{2}} \right)^{2} }}} }}$$

As noted above, for an exciton with a light hole, this quantization regime was not considered even in the case of the parabolic dispersion law, hence let us consider the differences from the Kane’s dispersion law in more detail. Using the coordinates of the center-of-gravity and relative motion, the Schrödinger equation for this case is written as:

47 $$\left( { - \frac{{\hbar^{2} }}{{2M_{0} }}\nabla_{{\vec{R}}}^{2} - \frac{{\hbar^{2} }}{2\mu }\nabla_{{\vec{r}}}^{2} + \frac{{e^{2} }}{{\kappa \left| {\vec{r}} \right|}}} \right)\psi_{{n_{r} ,m,n_{z} }} \left( {\vec{r}} \right)\Phi_{{n_{Gr} ,n_{R} ,M}} \left( {\vec{R}} \right) = E\,\psi_{{n_{r} ,m,n_{z} }} \left( {\vec{r}} \right)\Phi_{{n_{Gr} ,n_{R} ,M}} \left( {\vec{R}} \right)$$

In the $E_{ex}$ and $a_{ex}$ units Eq. (47) will be written in the form:

48 $$\left( { - \frac{1}{4}\nabla_{{\vec{R}}}^{2} - \nabla_{{\vec{r}}}^{2} - \frac{2}{{\left| {\vec{r}} \right|}}} \right)\psi_{{n_{r} ,m,n_{z} }} \left( {\vec{r}} \right)\Phi_{{n_{Gr} ,n_{R} ,M}} \left( {\vec{R}} \right) = \varepsilon \,\psi_{{n_{r} ,m,n_{z} }} \left( {\vec{r}} \right)\Phi_{{n_{Gr} ,n_{R} ,M}} \left( {\vec{R}} \right)$$

Separating the variables and solving the Schrödinger equation, as in the case of the Kane’s dispersion law, for the center-of-gravity energy one obtains the result (28). However, for radial motion, in the case of the parabolic dispersion law, one gets

49 $$\left( {\frac{{\partial^{2} }}{{\partial z^{2} }} + \frac{{\partial^{2} }}{{\partial r^{2} }} + \frac{1}{r}\frac{\partial }{\partial r} + \frac{1}{{r^{2} }}\frac{{\partial^{2} }}{{\partial \varphi^{2} }} + \left( {\varepsilon_{0} - \varepsilon_{Gr} } \right) + \frac{2}{r}} \right)\psi_{{n_{r} ,m,n_{z} }} \left( {r,\varphi ,z} \right) = 0$$

Note that Eq. (49) is also obtained considering the condition $a_{ex} > > c_{1}$ and omitting the terms proportional to $\frac{{z^{2} }}{{r^{2} }} \to 0$. Again the WFs are sought in the form (40) and for the motion in the $OZ$-direction, repeating the calculation procedure, one obtains the results (41) and (42). However, for the radial part, in this case, an equation similar to the 2D Coulomb equation is obtained:

50 $${\rm X}^{\prime\prime}\left( r \right) + \frac{1}{r}{\rm X}^{\prime}\left( r \right) + \left( {\varepsilon_{2} - \frac{{m^{2} }}{{r^{2} }} + \frac{2}{r}} \right){\rm X}\left( r \right) = 0$$

the solutions of which are given by degenerate hypergeometric functions of the first kind:

51 $${\rm X}\left( \xi \right) = \xi^{\left| m \right|} e^{{ - {\raise0.7ex\hbox{$\xi $} \!\mathord{\left/ {\vphantom {\xi 2}}\right.\kern-\nulldelimiterspace} \!\lower0.7ex\hbox{$2$}}}}_{1} F_{1} \left( { - \left( {\frac{1}{{\sqrt { - \varepsilon_{2} } }} - \left| m \right| - \frac{1}{2}} \right),2\left| m \right| + 1,\xi } \right)$$

A similar result for the case of the parabolic dispersion law is written as:

52 $$\varepsilon_{int}^{Par} = \frac{{\pi^{2} n_{Gr}^{2} }}{{16c^{2} }} + \frac{{\pi n_{Gr} }}{4ac}\left( {2n_{R} + \left| M \right| + 1} \right) + \frac{{\pi^{2} n_{z}^{2} a^{2} }}{{4c^{2} \left( {a^{2} - 1} \right)}} - \frac{1}{{\left( {N_{C} + \frac{1}{2}} \right)^{2} }}$$

where $N_{C} = n_{r} + \left| m \right|$ is Coulomb principal QN for exciton.

It is also important to make the following remarks here:

- In contrast to the 3D exciton case, all states with $m = 0$ are unstable in a semiconductor with Kane’s dispersion law. It is also important that instability is the consequence not only of the dimension reduction of the sample, but also the change in the dispersion law. “The particle falling into center” or the recombination (exciton collapse) of the pair in the states with $m = 0$, is the consequence of interaction of energy bands. Thus, the dimension reduction leads to the fourfold increase in the exciton ground state energy in case of parabolic dispersion law, but in the case of Kane’s dispersion law, recombination (exciton spontaneous collapse) is also possible. Note also that the presence of quantum confinement does not affect the occurrence of instability, as it exists in both the presence and absence of quantum confinement (see formulae above).
- Consideration of the bands’ interaction removes the degeneracy of the magnetic QN. However, the twofold degeneracy of $m$ of energy remains. Thus, in the case of Kane dispersion law the exciton energy depends on $m^{2}$, whereas in the parabolic case it depends on $\left| m \right|$. Due to the circular symmetry of the problem, the twofold degeneracy of energy holds in both cases of dispersion law.
- In this quantum confinement regime, the behavior of the Coulomb levels is similar to the case of the weak quantum confinement regime. Thus, in the case of the implementation of the Kane’s dispersion law, the Coulomb interaction appears as a mixed interference term with the quantum confinement in the total energy, while in the case of a parabolic dispersion law, the Coulomb energy appears as an additive term (compare to 46 and 52).

## Interband Absorption of Light

Direct interband absorption of light in a strongly flattened ellipsoidal QD in a **strong quantum confinement regime** is considered, when the Coulomb interaction between the electron and a hole is neglected. The case of a light hole is discussed ($m_{e}^{ * } = m_{h}^{ * }$), and the absorption coefficient is determined by the expression [34]:

53 $$K = A\sum\limits_{{\nu ,\nu^{\prime}}} {\left| {\int {\Psi_{\nu }^{e} \Psi_{{\nu^{\prime}}}^{h} d\vec{r}} } \right|}^{2} \delta \left( {\hbar \Omega - E_{g} - E_{\nu }^{e} - E_{{\nu^{\prime}}}^{h} } \right)$$

where $\nu$ and $\nu^{\prime}$ are the QN sets corresponding to the electron and hole, $E_{g}$ is the band gap of a bulk semiconductor, $\Omega$ is an incident light frequency, $A$ is a quantity proportional to the square of the matrix element taken by the Bloch functions. After simple calculations, one gets the following expression for the absorption edge (AE) $W_{100}$:

54 $$W_{{Str}}^{{Par_{{100}} }} = 1 + \frac{{\pi ^{2} }}{4}\frac{{d^{2} }}{{c^{2} }} + \pi \frac{{d^{2} }}{{a\;c}}$$

where $W_{100} = \frac{{\hbar \Omega_{100} }}{{E_{g} }}$, $d = \frac{\hbar }{{\sqrt {2\mu E_{g} } }}$, $\mu = \frac{{m_{e}^{ * } m_{h}^{ * } }}{{m_{e}^{ * } + m_{h}^{ * } }}$—is the reduced mass of the exciton. For the Kane’s dispersion law

55 $$W_{{Str}}^{{Kane_{{100}} }} = \sqrt {1 + \frac{{\pi ^{2} d^{2} }}{{c^{2} }} + \frac{{8\pi d^{2} }}{{ac}}}$$

The selection rules in quantum transitions are considered. Quantum transitions for the energy levels allowed for the magnetic QNs $m = - m^{\prime}$, and for the "fast" subsystem QNs $n = n^{\prime}$, while the "slow" subsystem selection rules are $N = N^{\prime}$.

Next, let us consider the interband absorption of light in the weak quantum confinement regime. Due to the localization of the exciton in a relatively small vicinity of the geometric center of the QD, the expression for the absorption coefficient can be written as [34]

56 $$K = A\sum\limits_{\begin{subarray}{l} n_{Gr} ,n_{R} ,M \\ \,\,\,\,\,\,\,\,\,\,n_{r} ,l \end{subarray} } {\left| {\psi_{{n_{r} ,l,q}} \left( 0 \right)} \right|^{2} \left| {\int {\Phi_{{n_{Gr} ,n_{R} ,M}} \left( {\vec{R}} \right)\Phi_{{n,n_{r} ,m}} \left( {\vec{R}} \right)d\vec{R}} } \right|}^{2} \delta \left( {\hbar \Omega - E_{g} - E} \right)$$

where $E$ is the energy (37) in dimensional units. It should be noted that $\psi_{{n_{r} ,l,q}} \left( 0 \right) \ne 0$ only for the ground state, when $l = q = 0$ ($l,q$ are the orbital and magnetic QNs of the exciton). In this regime, the following analytical expressions are finally obtained for the absorption coefficient and the AE:

57 $$K = A\sum\limits_{{n_{Gr} ,n_{R} }} {\frac{{32a_{1} c_{1}^{2} }}{{\pi^{5} n^{3} \left( {a_{ex} } \right)^{3} }}\frac{{n_{r} !}}{{\Gamma^{3} \left( {1 + n_{r} } \right)}}} {\kern 1pt} \delta \left( {\hbar \Omega - E_{g} - E} \right)$$

58 $$W_{weak}^{Par} {\kern 1pt}_{1001} = 1 + \frac{{\pi^{2} }}{16}\frac{{h^{2} }}{{c^{2} }} + \frac{{\pi h^{2} }}{4a\,c} - h^{2}$$

where $n_{r}$ is the radial QN of the exciton, $W_{1001\left( 0 \right)} = \frac{{\hbar \Omega_{1001\left( 0 \right)} }}{{E_{g} }}$, $h = \frac{\hbar }{{\sqrt {2M_{0} E_{g} } }}$, and $M_{0}$ is the total mass of the exciton. For the Kane’s dispersion law in the case of weak quantum confinement, one has

59 $$W_{{weak}}^{{Kane_{{1000}} }} = 1 - \frac{{\sqrt {\frac{1}{2} + \frac{{\pi ^{2} h^{2} }}{{16c^{2} }} + \frac{{\pi h^{2} }}{{4a{\mkern 1mu} c}}} }}{{\sqrt {1 + \frac{{4h^{2} }}{{\left( {\sqrt {\frac{1}{4} - 4h^{2} } + \frac{1}{2}} \right)^{2} }}} }}$$

and quantum transitions for the energy levels are allowed for the exciton radial QNs $n_{r} = n^{\prime}_{r}$ and $n_{r} = n^{\prime}_{r} \pm 1$, and for the "fast" subsystem QNs $n_{Gr} = n^{\prime}_{Gr}$, while the "slow" subsystem selection rules are $N = N^{\prime}$. In the case of the parabolic dispersion law, quantum transitions for the energy levels allowed for the exciton main QN $N_{C} = N^{\prime}_{C}$, while for the motion of center-of-gravity selection rules remain the same.

The most important feature of the latter case is that the shift of the exciton level with a change in the semiaxes of the QD is determined by the total mass of the exciton.

Let us proceed to consideration of direct interband light absorption in the QD in the intermediate quantum confinement regime. Here, the electron–hole interaction leads to the fact that in the spectrum of interband optical absorption, each line corresponding to specified values of $\nu$ turns into a series of closely spaced lines corresponding to different values of $\nu^{\prime}$. The absorption coefficient in this regime has the form [34]

60 $$K = A\sum\limits_{{\nu ,\nu^{\prime}}} {\left| {\Psi \left( {\vec{r}_{e} ,\vec{r}_{h} } \right)\delta \left( {\vec{r}_{e} - \vec{r}_{h} } \right)d\vec{r}_{e} d\vec{r}_{h} } \right|}^{2} \delta \left( {\hbar \Omega - E_{g} - E_{\nu }^{e} - E_{{\nu^{\prime}}}^{h} } \right)$$

Finally, in the case of the CC’s parabolic dispersion law, for the AE one obtains

61 $$W_{{int}}^{{Par_{{1010}} }} = 1 + \frac{{\pi ^{2} d^{2} }}{{16c^{2} }} + \frac{{\pi d^{2} }}{{4 ac}} + \frac{{\pi ^{2} a^{2} d^{2} }}{{4c^{2} \left( {a^{2} - 1} \right)}} - 4d^{2}$$

For the Kane’s dispersion law,

62 $$W_{{\text{int} }}^{{Kane_{{10101}} }} = 1 - \frac{{\sqrt {\frac{1}{2} + \frac{{\pi ^{2} d^{2} }}{{16c^{2} }} + \frac{{\pi d^{2} }}{{4ac}} + \frac{{\pi ^{2} a^{2} d^{2} }}{{4c^{2} \left( {a^{2} - 1} \right)}}} }}{{\sqrt {1 + \frac{{4d^{2} }}{{\left( {\sqrt {1 - \frac{4}{{\varepsilon _{g} }}} + \frac{1}{2}} \right)^{2} }}} }}$$

Strictly speaking, formula (62) is the reduced AE, since the exciton in the ground state with a QN $m = 0$ is not stable and collapses spontaneously. Therefore, this formula is written for the first stable excited state of an exciton with the magnetic QN $m = 1$. In the intermediate quantum confinement regime, for the case of the Kane’s dispersion law of CCs, quantum transitions are allowed between levels with $n_{r} = n^{\prime}_{r}$, $m = - m^{\prime}$ and $n_{z} = n^{\prime}_{z}$ exciton QNs, while for the motion of center-of-gravity selection rules are $n_{Gr} = n^{\prime}_{Gr}$ and $N = N^{\prime}$. In the case of parabolic dispersion law, quantum transitions for the energy levels allowed for the exciton main QN $N_{C} = N^{\prime}_{C}$ and $n_{z} = n^{\prime}_{z}$, while for the motion of center of gravity selection rules remain the same. Remarkably, in contrast to the case of the weak quantum confinement regime, for both cases of the dispersion law in the intermediate quantum confinement regime, the shift of the exciton level with a change in the geometric parameters of the QD is determined by the reduced exciton mass. Moreover, the superimposed effect of the quantum confinement and the Coulomb interaction of an electron and a hole leads to the emergence of new selection rules $n_{z} = n^{\prime}_{z}$ as a result of the vertical motion of the center-of-gravity with the reduced mass of the 2D exciton.

## Discussion

### Strong Quantum Confinement Regime

**Fig. 2:**
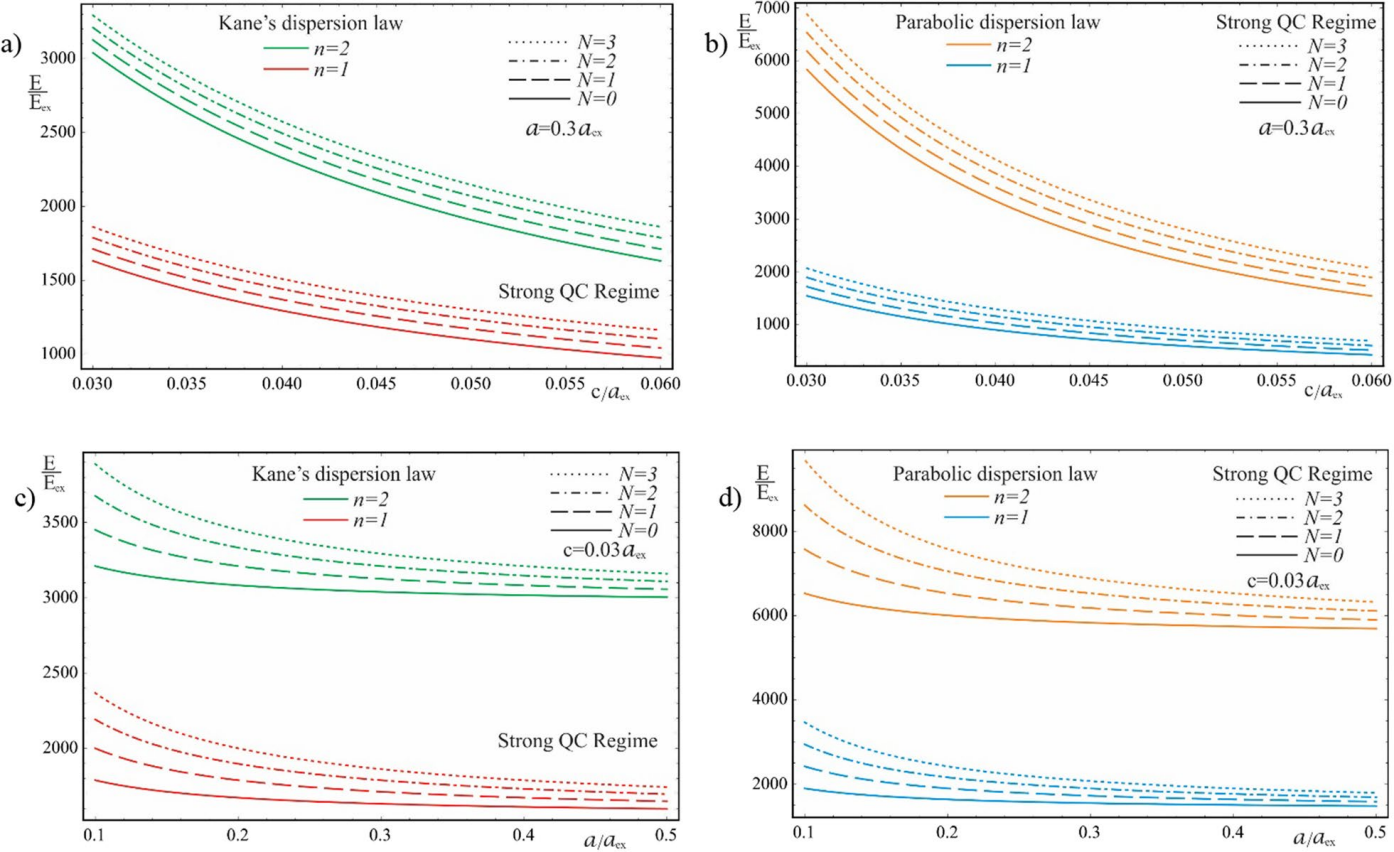
The dependences of the lower two families of levels of the energy spectrum of an electron in a strongly flattened ellipsoidal QD with the Kane’s and parabolic dispersion laws of CCs: (**a**) and (**b**) on the minor semiaxis $c$ at the fixed value of the major semiaxis $a$, (**c**) and (**d**) on the major semiaxis $a$ at the fixed value of the minor semiaxis *c*

**Fig. 3:**
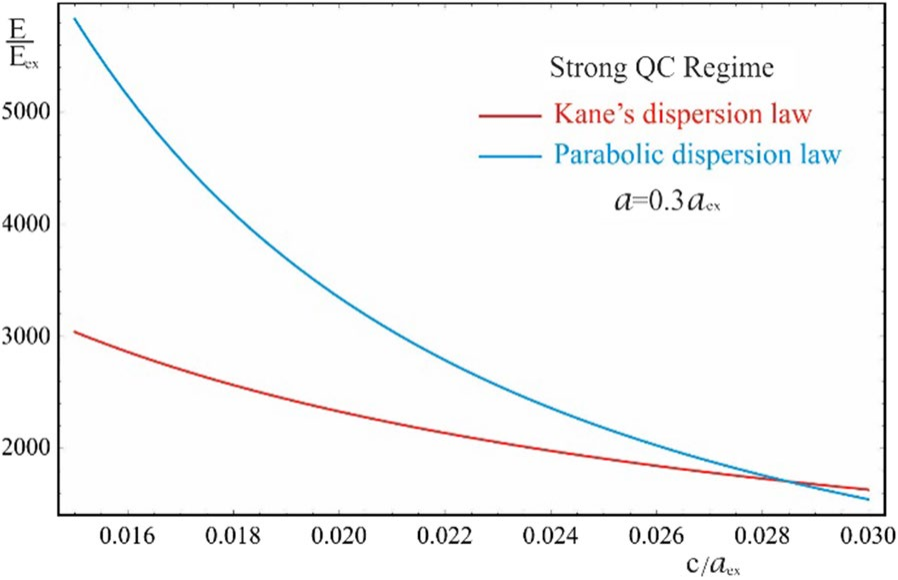
The dependence of the ground state energy on the minor semiaxis $c$ at the fixed value of the major semiaxis $a$ for the Kane’s and parabolic dispersion laws of CCs

**Fig. 4:**
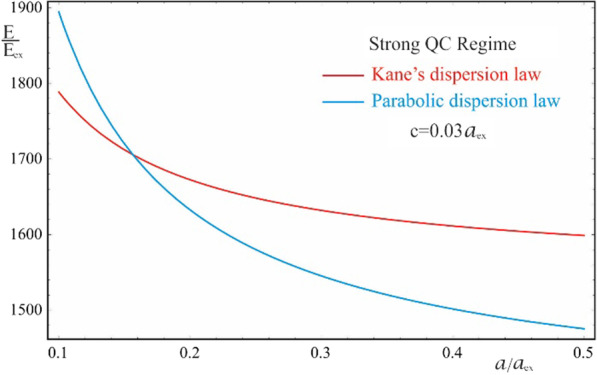
The dependence of the ground state energy on the major semiaxis $a$ at the fixed value of the minor semiaxis $c$ for the Kane’s and parabolic dispersion laws of CCs

**Fig. 5:**
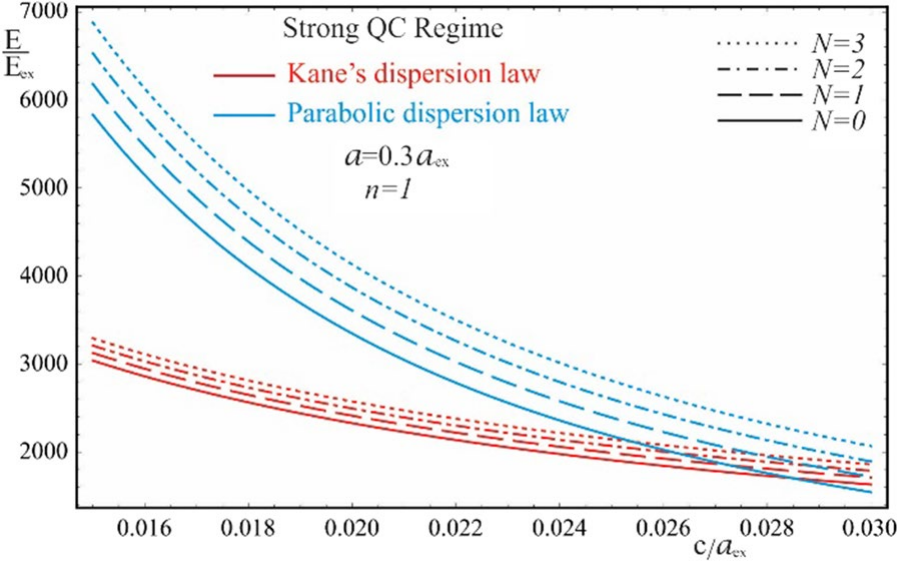
The dependence of the first family of the energy levels on the minor semiaxis $c$ at the fixed value of the major semiaxis $a$ for the Kane’s and parabolic dispersion laws of CCs

**Fig. 6:**
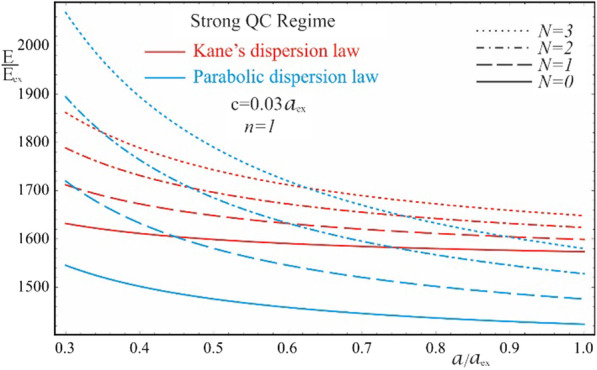
The dependence of the first family of the energy levels on the major semiaxis $a$ at the fixed value of the minor semiaxis $c$ for the Kane’s and parabolic dispersion laws of CCs

As is seen from the results obtained in (20) and (21), the energy spectra of particles corresponding to both the Kane’s and parabolic dispersion laws of CCs have a common characteristic. In both cases, for the lower levels of the spectrum, families of energy levels of the “slow” subsystem are located above each level of the “fast” subsystem, the interlevel distances of which depend on the QN $n\left( {n^{\prime}} \right)$ of the “fast” subsystem. Figure 2 shows the dependences of the lower two families of levels of the energy spectrum of an electron in a strongly flattened ellipsoidal QD with Kane’s and parabolic dispersion laws of CCs. Numerical calculations are made for the QD consisting of $InSb$ with the following parameters: $m_{e}^{*} = m_{h}^{*} \simeq 0.013m_{0}$, $E_{ex} \simeq 3 \cdot 10^{ - 4} eV$, $E_{g} \simeq 0.23\,eV$, $\kappa = 17.8$, $a_{{ex}} \simeq 10^{3}$ Å, where $m_{0}$ is the mass of the free electron. As can be seen from the figure, with an increase in the semiaxes, the energy levels in the case of both dispersion laws decrease due to a decrease in the influence of quantum confinement. As expected, with an increase in the semiminor axis $c$, the levels decrease more sharply than with an increase in the semi-major axis $a$ (compare graphs (a) and (c), and graphs (b) and (d) in Fig. 2), since the quantum confinement in the perpendicular direction has a much greater contribution to the energy of the particle than the quantum confinement in the radial direction, due to the strongly flattened external shape of the ellipsoidal QD. However, in the case of the parabolic dispersion law, the families of the “slow” subsystems turn out to be equidistant, while in the case of the Kane’s dispersion law, equidistance is violated. Thus, for the case of the Kane’s dispersion law with the values of the semiaxes $a = 0.3a_{ex}$ and $c = 0.05a_{ex}$ (see Fig. 2a), for the first family of levels ($n = 1$) we have the following interlevel distances: $E_{1,1}^{Kane} - E_{1,0}^{Kane} \simeq 70.68E_{ex}$, $E_{1,2}^{Kane} - E_{1,1}^{Kane} \simeq 66.65E_{ex}$, and $E_{1,3}^{Kane} - E_{1,2}^{Kane} \simeq 63.24E_{ex}$. For the second family of levels ($n = 2$), the following interlevel distances are obtained: $E_{2,1}^{Kane} - E_{1,0}^{Kane} \simeq 82.36E_{ex}$, $E_{2,2}^{Kane} - E_{2,1}^{Kane} \simeq 79.08E_{ex}$, and $E_{2,3}^{Kane} - E_{2,2}^{Kane} \simeq 76.18E_{ex}$. For the same values of the semiaxes, in the case of the parabolic dispersion law (see Fig. 2b), for all levels of the first family one obtains the result $\Delta E_{1}^{Par} \simeq 104.72E_{ex}$, and for the second family, as expected, all interlevel distances are twice as large: $\Delta E_{2}^{Par} \simeq 209.44E_{ex}$. As can be seen from these data, the frequencies corresponding to the energies of interlevel distances in both cases of the CCs’ dispersion law belong to the infrared spectrum. Thus, for the values of the semiaxes $a = 0.3a_{ex}$ and $c = 0.05a_{ex}$, in the case of the Kane’s dispersion law, for the first interlevel transition, the frequency $\omega = 3.22 \times 10^{13} Hz$ is obtained, and for the parabolic dispersion law, $\omega = 4.77 \times 10^{13} Hz$ is obtained. For the second family of energies, the frequencies remain in the infrared range as well. For instance, $\omega = 0.95 \times 10^{14} Hz$ for the parabolic dispersion law, whereas the values are smaller for the case of the Kane’s dispassion law. As is known, for many diatomic molecules, the frequencies corresponding to vibrational modes fall into the infrared region $\omega \sim 10^{13} \div 10^{14} Hz$ [35–37]. The typical vibrational frequencies range from less than $10^{13} Hz$ to approximately $10^{14} Hz$, corresponding to wavenumbers of approximately 300–3000 cm^−1^ and wavelengths of approximately 30–3 µm. At low ambient temperatures, vibrational modes of molecules with high accuracy can be approximated by harmonic oscillations. In other words, the vibrational levels of the molecules are quasi-equidistant. At high temperatures, which correspond to room temperatures and higher, the anharmonicity of the oscillations becomes significant, and, therefore, the equidistance of the vibration levels is violated. Moreover, for polyatomic molecules, vibrational modes can be both symmetric and asymmetric, hence both anharmonicity and asymmetry of vibrations need to be considered. For detecting such vibrations, strongly flattened ellipsoidal QDs with the Kane’s dispersion law are excellently suited, since their energy levels, due to their non-equidistance, can easily mimic the vibrational levels of complex molecules. For simpler diatomic molecules, or for lower temperatures, it is preferable to use QDs with the parabolic dispersion law. Since their energy levels are equidistant, with an appropriate choice of geometric parameters, they are perfect for mimicking the spectra of diatomic molecules. Figure 3 shows the dependence of the ground state energy on the minor semiaxis $c$ at the fixed value of the major semiaxis $a$ for both cases of CC’s dispersion laws, and Fig. 4 shows a similar dependence, but only on the major semiaxis $a$ at the fixed value of the minor semiaxis $c$. As can be seen from the figures, as the values of the semiaxes increase, the energy curves corresponding to different dispersion laws intersect. In both cases, at relatively small values of the semiaxis, the particle energy curve corresponding to the case of the parabolic dispersion law is positioned higher than the curve corresponding to the two-band Kane’s dispersion law. With an increase in the values of the semiaxes, the curves change places. This happens because the particle energy in the case of a parabolic dispersion law is proportional to $\sim \frac{1}{{c^{2} }}$ or $\sim \frac{1}{ac}$, while in the case of the two-band Kane’s approximation, an analogous proportionality appears under the square root sign (see 20). It is for this reason that the energy of a particle in the parabolic case turns out to be larger for small values of the semiaxes and is less than the energy of the Kane’s dispersion law case for large values of the semiaxes. The intersection point means that the energies of the two cases of the CC’s dispersion law are the same. However, this does not mean the identity of the entire spectrum. Dependence of the first family of levels of the energy spectrum of an electron in a QD with Kane’s and parabolic dispersion laws on the semiminor axis is shown on Fig. 5. As can be seen from the figure, the energy levels corresponding to different values of QNs intersect at different values of the semiaxes. A similar picture is observed in Fig. 6 which shows the dependence of the first family of levels of the energy spectrum of an electron on the semi-major axis for both dispersion laws. It should be noted that in both figures, with an increase in the QN value, the intersection point of the energy levels shifts to the right (toward the large values of the semiaxes) due to the above reason.

**Fig. 7:**
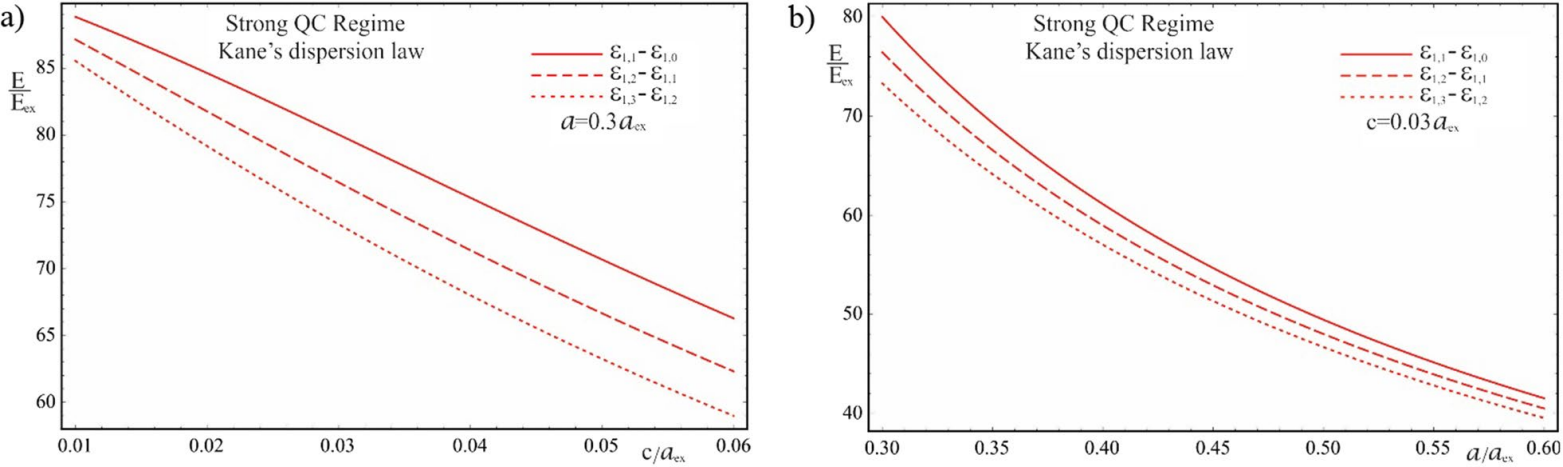
The dependence of the interlevel distances of the first family of electron energy levels on the semiaxes of the ellipsoidal QD in the case of a complicated (Kane’s) dispersion law: (**a**) on the minor semiaxis $c$ at the fixed value of the major semiaxis $a$, (**b**) on the major semiaxis $a$ at the fixed value of the minor semiaxis

Figure 7 shows the dependence of the interlevel distances of the first family of electron energy levels on the semiaxes of the ellipsoidal QD in the case of a complicated Kane’s dispersion law. As can be seen from the graph, with an increase in the values of both semiaxes, the interlevel distances of the particle energy decrease by a nonlinear dependence, and interlevel distances are different for different sets of QNs. Hence, by changing the geometric parameters of the QD, the desired distances of the levels within the same family can be obtained much more flexibly than in the case of the parabolic dispersion law, where the interlevel distances within each family vary the same depending on the values of the semiaxes. Thus, it can be stated that for the strong quantum confinement regime, in the case of using a QD with the Kane’s dispersion law, manipulating interlevel distances allows covering an even larger number of possible analyte molecules, which can be detected by mimicking their vibration levels. Obviously, the more flexible possibility of controlling the interlevel distances within each family increases the probability of successful electron tunneling between analytes and QDs, and their registration.

### Weak Quantum Confinement Regime

Let us proceed to discussing the results of the weak quantum confinement regime, when the energy of the Coulomb interaction of an electron and a hole exceeds the quantum confinement energy in all three geometric directions. The formation of an exciton and the quantization of its motion as a whole dramatically changes the energy spectrum of a QD. An important feature of the implementation of this regime is the ability to cover new energy areas for the purposes one pursues, in particular, negative energies. As can be seen from result (38), in the parabolic dispersion law case, the energy conditioned by quantum confinement of the QD walls is positive, and the Coulomb energy is negative. With a change in the geometric parameters of the QD, the total energy can change sign. The situation changes drastically in the Kane’s dispersion law case, where the energy is entirely negative (see 37). In the weak quantum confinement regime, consideration of the interband interaction in the case of the two-band Kane’s model leads to the superimposed manifestation of the Coulomb and quantum confinement energies. Moreover, the Coulomb energy in the Kane’s model case has an explicit dependence on the radial and orbital QNs. The interband interaction in the case of the two-band model violates the high order of hidden symmetry of the Coulomb interaction due to the imposition of lower order symmetry [38]. Obviously, this cannot be a consequence of the imposition of the ellipsoidal symmetry of the QD itself, since the hidden symmetry is not violated in the case of the parabolic dispersion law. In both cases of dispersion laws in the weak quantum confinement regime, a 3D exciton with spherical symmetry is formed. The ellipsoidal shape (symmetry) of the QD affects only the quantization of the motion of the exciton's center-of-gravity, while not breaking the internal spherical symmetry of the exciton. Internal symmetry is broken (lowered) due to the interaction of the bands and is a characteristic of a narrow-gap semiconductor material. Such a complex interaction is absent in the case of a parabolic dispersion law, which is the main feature of the two-band Kane’s model.

**Fig. 8:**
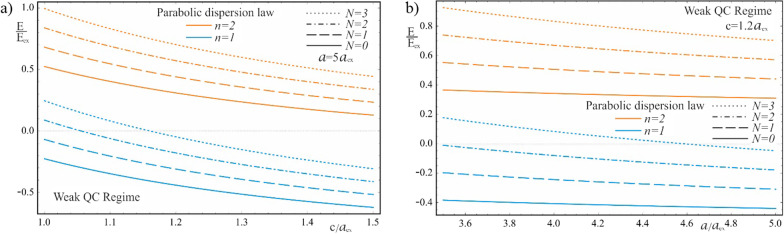
The dependences of the exciton energy on (**a**) the minor semiaxis $c$ at the fixed value of the major semiaxis $a$, (**b**) the major semiaxis $a$ at the fixed value of the minor semiaxis $c$ (parabolic dispersion law)

**Fig. 9:**
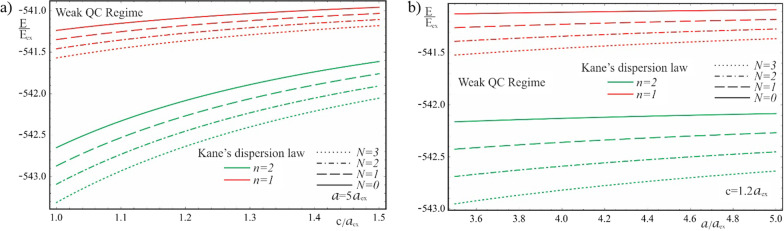
The dependences of the exciton energy on (**a**) the minor semiaxis $c$ at the fixed value of the major semiaxis $a$, (**b**) the major semiaxis $a$ at the fixed value of the minor semiaxis $c$ (Kane’s dispersion law)

Figure 8 shows the dependences of the exciton energy on the geometric parameters of the QD in the case of the parabolic dispersion law of CCs. As can be seen from the figure, with an increase in the values of both semiaxes, the energies decrease and shift to the region of negative energies. This is due to the fact that with a decrease in the influence of the quantum confinement, due to an increase in the size of QDs, the dominance of the negative Coulomb energy appears more distinctly. An unexpected inverse pattern of the dependence of the exciton energy on the geometric parameters of the QD is observed in the case of a complex two-band model dispersion law, which is shown in Fig. 9. With an increase in the values of the semiaxes, the quantum confined exciton energy increases (decreases in absolute value), despite the decrease in the effect of the QD walls. As expected, the dependence on geometric parameters is much weaker than in the case of a strong quantum confinement regime. As can be seen from the figure, the energy levels of the exciton are negative and are positioned much lower in contrast to the parabolic case. By absolute value, the energy of the Kane’s exciton is much higher than the exciton energy of the parabolic case. Thus, for the values of the semiaxes $a = 5a_{ex} ,\,\,c = 1.2a_{ex}$, the energy of the ground state of the exciton in the parabolic case is $\varepsilon_{weak}^{Par} \simeq 0.44E_{ex}$, while in the Kane’s case $\varepsilon_{weak}^{Kane} \simeq 541.08E_{ex}$. Comparison of the energy spectra (37) and (38) reveals another significant detail. At a fixed value of the QNs $n_{Gr}$ and $N_{R}$ and an increase in the value of the Coulomb QN $N_{C}$ leads to a shift of the excited Coulomb energy levels toward positive energies. The opposite shift of the Coulomb levels is observed in the case of the Kane’s dispersion law, when the excited Coulomb levels are shifted toward negative energies and are positioned lower. As noted above, in the parabolic case, the behavior of exciton levels is similar to that of donor levels, while in the Kane’s case it resembles the behavior of acceptor levels. It is noteworthy that in both cases of dispersions, the energy increases in absolute value with an increase in Coulomb QNs.

### Intermediate Quantum Confinement Regime

**Fig. 10:**
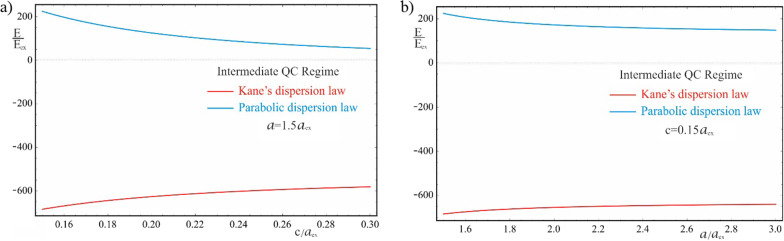
The dependences of the ground and first excited energy levels of a 2D exciton with parabolic and Kane’s dispersion laws of CCs, respectively, at the intermediate quantum confinement regime: (**a**) on the minor semiaxis $c$ at the fixed value of the major semiaxis $a$, (**b**) on the major semiaxis $a$ at the fixed value of the minor semiaxis *c*

Figure 10 represents the dependences of the ground and first excited energy levels of a 2D exciton on the geometric parameters of the QD, with parabolic and Kane’s dispersion laws of CCs, respectively. In the case of the Kane’s dispersion law, the ground level of the 2D exciton is unstable—it collapses spontaneously; therefore, the dependences of the first excited energy level are presented. As can be seen from the figure, the exciton energy is negative for the Kane’s dispersion law, as in the case of the weak quantum confinement regime, but, in contrast, the exciton energy does not change sign in the parabolic case. With an increase in the values of the semiaxes, the energy decreases due to a decrease in the contribution of the quantum confinement, but remains positive even for the ground state, due to the presence of a strong influence of the quantum confinement in the vertical direction. In the intermediate quantum confinement regime, due to the condition $c < < a_{ex}$, the value of the semiminor axis changes in such a range that the positive contribution to the energy from the quantum confinement in the vertical direction exceeds by an order of magnitude the negative contribution from the quasi-2D Coulomb interaction of the exciton. Hence, at the values of the semiaxes $a = 1.5a_{ex}$ and $c = 0.15a_{ex}$, the energy is $E_{int}^{Par} \simeq 224.3E_{ex}$, while at $c = 0.3a_{ex}$, $E_{int}^{Par} \simeq 53.95E_{ex}$. A twofold increase in the value of the semiminor axis leads to a sharp decrease in energy, but does not change its sign. The presence of a strong quantum confinement in the vertical direction also leads to a much larger difference between the energies of the Kane’s and parabolic cases, in contrast to the weak quantum confinement regime. Another feature of the implementation of the intermediate quantum confinement regime is the appearance of new energy levels due to the vertical quantization of the motion of the quasi-2D exciton. Also, in contrast to the weak quantum confinement regime, the motion of the center-of-gravity of a 2D exciton is conditioned by its reduced mass $\mu$, instead of the total mass $M_{0}$.

**Fig. 11:**
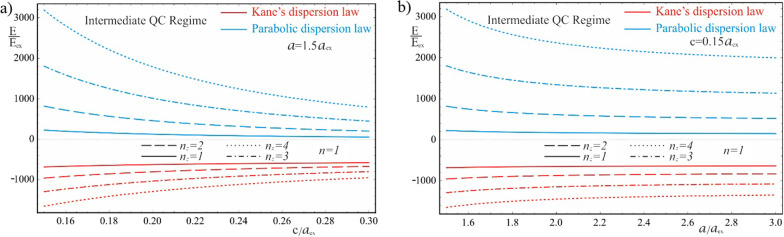
The dependence of the energy levels of vertical quantization of the motion of the center-of-gravity of a 2D exciton with parabolic and Kane’s dispersion laws of CCs: (**a**) on the minor semiaxis $c$ at the fixed value of the major semiaxis $a$, (**b**) on the major semiaxis $a$ at the fixed value of the minor semiaxis *c*

Figure 11 shows the dependence of the energy levels of vertical quantization of the motion of the center-of-gravity of a 2D exciton in a strongly flattened ellipsoidal QD with the Kane’s and parabolic dispersion laws of CCs. As can be seen from the figure, with an increase in the value of the vertical QN $n_{z}$, the interlevel distances of excited energy levels become very large, since the reduced mass $\mu$ is less than the total mass $M_{0}$ (for the *InSb* QD, $M_{0} = 4\mu$). Therefore, in contrast to the heavy 3D exciton in the weak quantum confinement regime, in the intermediate regime, the motion of a much lighter 2D exciton in the QD is quantized, which accounts for the large values of excited energies. The energy levels of the 2D exciton conditioned by the relative motion of an electron and a hole in the radial direction, are similar to the levels of a 3D exciton in the weak quantum confinement regime. For these levels, the analogy with donor and acceptor levels also takes place, as in the case of a weak quantum confinement regime. However, since the decrease in the dimension enhances the Coulomb interaction, the binding energy of a 2D exciton is always greater than the binding energy of a 3D exciton. Thus, in the case of a parabolic dispersion law, the binding energy of a 2D exciton is fourfold of a 3D exciton energy (compare the last terms in formulas (38) and (52), due to a decrease in dimensionality, and in both cases depends on the reduced mass $\mu$.

### Light Absorption

**Fig. 12:**
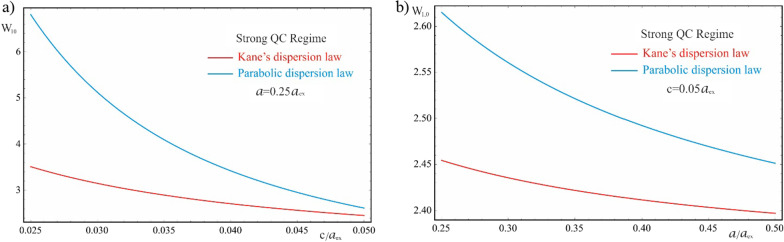
The dependences of the AE for Kane’s and parabolic dispersion laws of CCs in the strong quantum confinement regime: (**a**) on the minor semiaxis $c$ at the fixed value of the major semiaxis $a$, (**b**) on the major semiaxis $a$ at the fixed value of the minor semiaxis *c*

Figure 12 shows the dependences of the AE on the QD semiaxes for Kane’s and parabolic dispersion laws of CCs in the strong quantum confinement regime. For both dispersion laws, with an increase in the values of the semiaxes, the AE decreases due to a decrease in the contribution of the quantum confinement, since the “effective” band gap, $E_{g}$ plus the energy of quantum confined levels, decreases, and the absorption frequencies belong to the infrared range of the spectrum [39]. As can be seen from the graphs, the decrease in the AE is more distinct for the dependence on the semiminor axis. The AE curve corresponding to the Kane’s dispersion law is positioned lower (redshift) due to the weaker root dependence of the CC’s energy on the values of the QD’s geometric parameters. Therefore, for the Kane’s dispersion law, with an increase in the semiaxes, the decrease in the AE is pronounced weaker that in the case of parabolic dispersion, where the curve decreases more sharply.

**Fig. 13:**
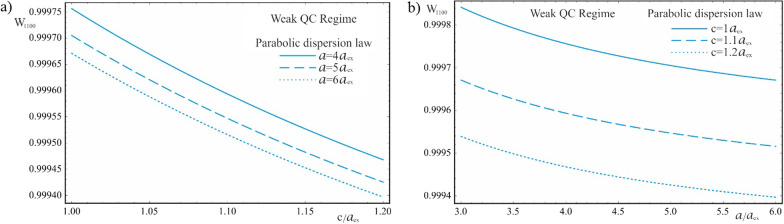
The dependences of the AE for the parabolic dispersion law of CCs in the weak quantum confinement regime: (**a**) on the minor semiaxis $c$ at the different values of the major semiaxis $a$, (**b**) on the major semiaxis $a$ at the different values of the minor semiaxis *c*

As is seen from Fig. 13, in the case of the parabolic dispersion also, for the weak quantum confinement regime, the AE dependence on the QD’s geometric parameters is more distinct for the dependence on the semiminor axis. For the same reason, the curves corresponding to different values of the semiminor axis are positioned with a greater relative shift (Fig. 13b) than in the opposite case (Fig. 13a). The Coulomb interaction consideration leads to a decrease in the width of the “effective” band gap and to the appearance of energy levels inside the forbidden band gap. Hence, the quantum transitions occur at lower values of the incident light frequency, shifting the AE toward the longer wavelengths.

**Fig. 14:**
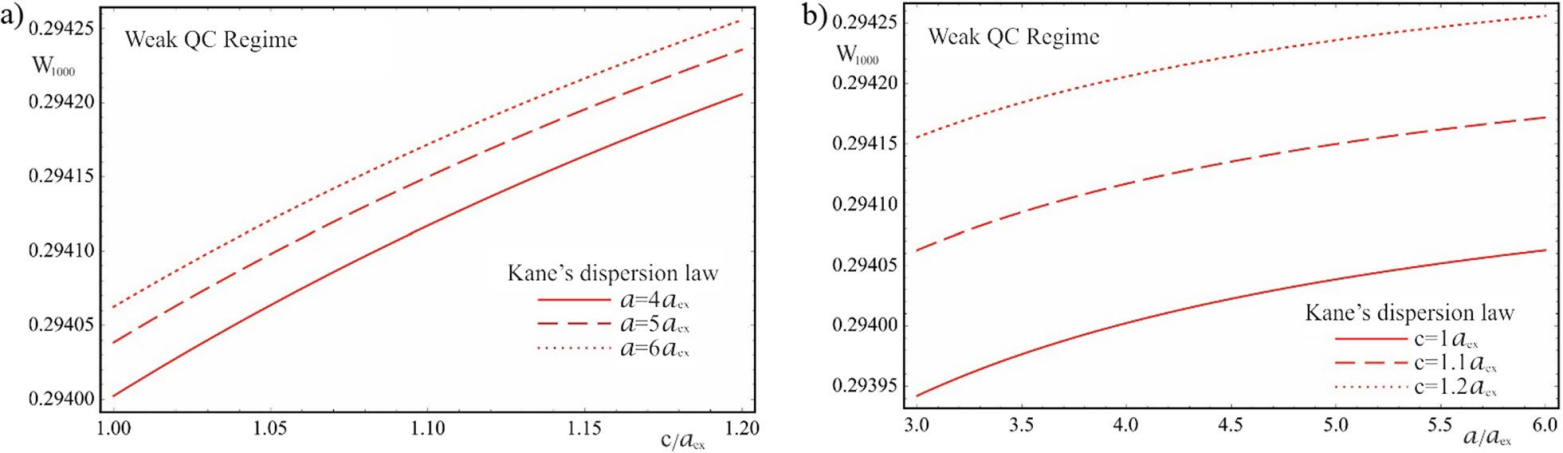
The dependences of the AE for the Kane’s dispersion law of CCs in the weak quantum confinement regime: (**a**) on the minor semiaxis $c$ at the different values of the major semiaxis $a$, (**b**) on the major semiaxis $a$ at the different values of the minor semiaxis $c$

Figure 14 shows the dependence of the AE on the QD’s geometric parameters for the Kane’s dispersion law in weak quantum confinement regime. The interband interaction leads to a significant shift of the AE frequencies toward the far infrared range, and the change in the AE appears more distinctly for the dependence on the semiminor axis. However, in contrast to the parabolic case, an increase in the QD’s geometric parameters leads to a blue shift of the threshold frequencies, as a result of a complex effect of quantum confinement and the Coulomb interaction along with interband interaction in the two-band Kane’s model. The curves corresponding to the large values of the semiaxes are shifted toward the shorter wavelengths (dashed lines on the graphs), which is the opposite behavior compared to the parabolic case.

**Fig. 15:**
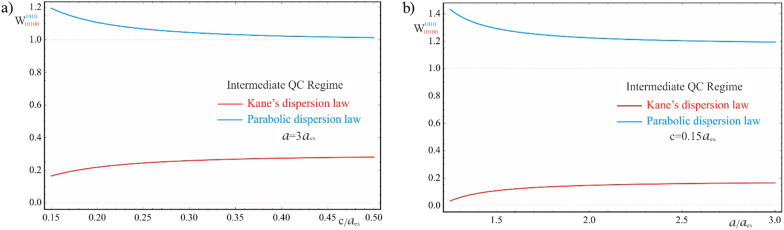
The dependences of the AE for Kane’s and parabolic dispersion laws of CCs in the intermediate quantum confinement regime: (**a**) on the minor semiaxis $c$ at the fixed value of the major semiaxis $a$, (**b**) on the major semiaxis $a$ at the fixed value of the minor semiaxis $c$

Finally, Fig. 15 represents the dependences of the AE in the intermediate quantum confinement regime on the geometric parameters of the QD for parabolic and Kane dispersion laws of CCs. As can be seen from the graphs, in the parabolic case, 2D Coulomb interaction does not sufficiently displace the AE toward longer waves, since even at the value of $c = 0.5a_{ex}$ of the semiminor axis, the absorption edge remains $W_{1010} > 1$. Hence, in contrast to the weak quantum confinement regime, the energy levels do not shift further in the forbidden band gap, and the width of the “effective” band gap is larger than $E_{g}$, due to the stronger contribution of the quantum confinement in the vertical direction. As in the case of the weak quantum confinement regime, in the intermediate quantum confinement regime with the Kane’s dispersion law, a shift of the threshold absorption frequencies toward short wavelengths is observed, in contrast to the parabolic case.

## Conclusion

In this work, the electronic and exciton states in a strongly flattened ellipsoidal QD have been considered in three quantum confinement regimes: strong, weak, and intermediate, both in the case of the two-band Kane’s dispersion law and the parabolic dispersion law of CCs. Analytical expressions for the WFs and for the energies of CCs have been obtained in a QD with a complex dispersion law of CCs in all regimes of the quantum confinement. The obtained results have been compared with the case of the CC’s parabolic law of dispersion. For a narrow-gap semiconductor, the possibility of spontaneous exciton collapse (instability) has been revealed for all levels with a QN $m = 0$ in the intermediate quantum confinement regime, while for a wide-gap semiconductor the levels are stable. The removal of an accidental Coulomb degeneracy of energy in the orbital QN for the 3D exciton in *InSb* QD and in a magnetic QN for the 2D exciton, as a result of a CC dispersion law symmetry degree reduction, has been noticed. It has been shown that in the strong quantum confinement regime, QDs with the Kane’s dispersion law are better suited for detecting vibrational levels of asymmetric molecules or vibrations at high temperatures, while QDs with the parabolic dispersion law are preferable for detecting vibrational levels of symmetric molecules or vibrations at low temperatures. Consideration of the Coulomb interaction and the possibility of the formation of 2D or 3D excitons have been shown to expand the range of encompassed energies for analyte detection. In particular, for the Kane’s dispersion law, a significant shift of energies to the region of negative energies has been revealed. The dependences of the AEs on the geometric parameters of the QD have been revealed, and the corresponding selection rules for quantum transitions have been obtained. New selection rules in the intermediate quantum confinement regime have been revealed. An atypical blue shift of the threshold absorption frequencies with an increase in the sample size for *InSb* QDs has been obtained due to a complex superimposed contribution of the interband and Coulomb interactions.

## Data Availability

Not applicable.

## Abbreviations

QD: Quantum dot
CC: Charge carrier
WF: Wave function
QN: Quantum number
AE: Absorption edge
